# Revelations of the Microscope

**Published:** 1874-05

**Authors:** 


### REVELATIONS OF THE MICROSCOPE.

*From a Paper by * * *.*

We are indebted to the persevering and laborious investigations of men of science for the development and elucidation of the principles which led to the construction of two most noble instruments, which must be ranked among the proudest triumphs of the intellect and skill of man—the telescope and microscope.

The numerous combinations of machinery that stand in the workshops of art, which, in obedience to the mysterious thought of the governing mind of man, perform their allotted task so perfectly that they seem to be almost gifted with intelligence, are all, at best, but instruments, by means of which we subject to our use powers and agencies long ago familiar to us. Although by their means we can apply to new purposes and new ends the resources that mankind has possessed for ages, and bring the physical world under our complete control, still, they are powerless to extend the limits of our knowledge or unfold one page in the great history of creation, ever reflecting the glorious attributes of divinity.

The portion of the universe with which we are conversant is as large without them as with them; they add no new regions to the field of knowledge nor reveal to our astonished gaze, worlds and existences, curious organizations, harmonies and forms of beauty hitherto unknown.

This is not the case with the telescope and microscope; they have a higher duty to fulfill; they are rather the companions than the slaves of their creator, ministering to the demands of his mind, rather than to the wants of his body. They accompany the flight of genius in its soarings through the regions of imagination, and enable it to give, to what may appear to others a wild and visionary conception, a reality and permanence as enduring as the universe itself; or they may take the lead, and on a sudden dazzle the beholder with visions of such rare magnificence and beauty, that truth surpasses fiction, and the fairy dreams of the imagination are more than realized.

By their means the boundaries of knowledge are extended; but the assistance that each renders in this respect is essentially different. The telescope reveals creations that lie beyond our globe; the microscope those that are within it, although too small to be seen with the naked eye. The former tells us of the nature and motions of the starry bodies, which in their undeviating march from century to century never grow weary or dim, but still beam upon us in all their primeval radiance and beauty, furnishing, as they do, one of the strongest proofs of a superior and all-ruling power.

It tells us that there are worlds and suns, and systems of suns, like our own, rushing with inconceivable speed through the illimitable fields of space, the superior orbs moving in the midst of a glittering zone of attendant worlds, yet each advancing in a fixed but invisible path, and guided in their course by laws as immutable as the word of Him who created them. At every progressive step the revelations become more amazing, and scene after scene of mysterious grandeur is successively revealed; but at the utmost verge of discovery we are still upon the threshold of creation, glimpses only of the infinite are beheld, and, far as the loftiest mind can soar, it perceives but a handbreadth of the splendid and imposing panorama of the skies. It is said that when, by the mighty power of the telescope, man was first enabled to gaze into space with the ken of an angel, and to recognize the orbs that glittered in the firmament as worlds like our own, countless in number, and stretching away through widening circles in all the vastness and magnificence of indefinitude, infidelity advanced once more to the attack, and argued against revelation from the immensity of creation, affirming that man was too insignificant to be the peculiar care of Him who had filled the illimitable regions of space with such stupendous works. Although this argument of infidelity is entirely false, it is, nevertheless, calculated to exercise a pernicious influence on weak and wavering minds, although, to those who are strong in faith, the condescension of the Almighty in regarding man at all has ever appeared amazing. Comparatively little was known in regard to the splendor of the universe in the time of the Psalmist, yet even he breaks forth in strains of admiration, as he lifts his eyes and beholds the moon and stars which illuminate the vast canopy of heavens: "When I consider Thy heavens, the work of Thy fingers; the moon and stars, which Thou hast ordained; what is man, that Thou art mindful of him?"

The microscope, in its revelations, advances in a directly opposite direction to that of the telescope; while the one delineates to us creations of infinite magnitude and extent, the other reveals to us creations which are infinitely small; and in these revelations the microscope serves not only to turn aside the attacks of infidelity, but causes them to be directed against its own weak armor; for by the assistance of this instrument we are enabled to follow the designs of divinity into worlds of creation so infinitely small that man himself, compared to them, is a world in magnitude and construction. Yet in these simple and apparently unimportant organizations, we find the workings of divine benevolence as visibly stamped as on the starry vault, glittering in the adamantine brilliancy of its rolling worlds. Here is impressed in our minds with new force the harmony of Nature with Revelation, and the truthfulness of the teaching, that not "a sparrow shall fall to the ground without the Father."

The microscope places us in the midst of a world which, before its discovery, was invisible, and without the assistance of which would ever have remained closed to our eyes.

Far as our assisted sight may pierce into this world, all is instinct with life, clothed in strange and curious forms, and replete with harmony, skill and wise design. Here our discoveries terminate, not for want of unknown fields to explore, but because our sight grows dim, and no further means are at our disposal for dispelling the darkness which gradually enshrouds the surrounding regions of infinitude.

By means of this instrument we learn that our accumulated mountains and extensive plains are composed of the shells of myriads of minute animals, which ages ago sported in the full activity of life; that the rocks quarried from these mountains of accumulated shells, form the basis of the solid and costly structures of our stately cities.

Here let us change the scene a little, and from the contemplation of the mountains and valleys glance into the water, whieh forms about two-thirds of the entire bulk of our earth. Let us take a drop of this clear and liquid fluid, place it under the object-glass of a powerful microscope. What visions of grandeur are suddenly opened up to our view! The drop of water is suddenly transformed into a vast ocean, teeming with life; curious forms are seen, sporting at will through its waters, apparently enjoying the rich endowments of life.

The microscope is also a powerful auxiliary to the skillful physiologist in his researches into the hidden mysteries of the vital system, and by this means, within a few years, much valuable information has been gained in regard to the curious processes of life, and the organization and wondrous mechanism of the human body.

By means of this instrument, one of the products of man's inventive genius, he is now enabled to gaze into the wonderful and mysterious picture of his own creation and development out of the small particle of primeval plasm. He finds, in his retrospective survey, that at a period in the early history of his development, he consists of but a congeries of cells, each of which has an independent existence of its own; going still further back, he finds that all of them have their origin in the subdivision of one primary cell, which is the first defined product of the generative act.

He finds that, by means of the various changes and evolutions which take place in these cells, are formed the different tissues, blood-vessels, nerves and organs, which collectively constitute the complex system of man.

By means of the microscope we are now enabled to witness the marvelous spectacle of the movement of the blood in the capillary vessels of the frog's foot, and thus verify by ocular demonstration the doctrine of the passage of the blood from the smallest arteries to the smallest vein, which had been propounded as a rational probability by the immortal Harvey.

But the fertile fields of knowledge opened up by the microscope in the history of the development of man leads the inquiring mind of the physiologist to proceed a step further in his investigations, and he pursues the like researches into the history of other living beings, and is soon led to the conclusion that the same is true of them also; every animal, every plant and in fact every organized thing in existence, has the same starting point in the single primeval cell; and it is thus in pursuing, by means of the microscope, the history of the organic germ from the most simple form of life to the most complex and intricate organism of man, that the physiologist is led to his grandest conception of the unity and all-comprehensive nature of the Almighty.

